# Personal

**Published:** 1893-10

**Authors:** 


### Personal.

At the recent meeting of the American Microscopical Society, held at Madison, Wis., Dr. Wm. C. Krauss, of Buffalo, was awarded the prize for the best collection of mounted slides.

Dr. A. Walter Suiter, of Herkimer, N. Y., ex-president of the Medical Society of the State of New York, received the degree of A. M. at the commencement of Union University, held in June, 1893.

Dr. H. C. Leonhardt, of Tonawanda, sailed for Europe on September 1, 1893, by the steamer Normanna. He will pass a year on the continent in perfecting himself in medical and surgical knowledge, first, however, spending several weeks in a general tour of observation and pleasure, visiting Germany, Switzerland, and Italy, after which he will locate for several months in Vienna.

